# Human-like maternal left-cradling bias in monkeys is altered by social pressure

**DOI:** 10.1038/s41598-020-68020-3

**Published:** 2020-07-03

**Authors:** Grégoire Boulinguez-Ambroise, Emmanuelle Pouydebat, Éloïse Disarbois, Adrien Meguerditchian

**Affiliations:** 1 Laboratoire de Psychologie Cognitive UMR7290, CNRS, Aix-Marseille Université, 3 Place Victor Hugo, 13331 Marseille Cedex 3, France; 2 Station de Primatologie, CNRS, Rousset-sur-Arc, France; 3 Mecanismes Adaptatifs et Évolution UMR 7179-CNRS, National Museum of Natural History, 75321 Paris Cedex 5, France

**Keywords:** Social behaviour, Biological anthropology, Evolutionary ecology, Animal behaviour

## Abstract

About 66–72% of human mothers cradle their infants on their left side. Given that left-cradling exposes the baby’s face to the mother’s left visual field (i.e., mainly projected to her right hemisphere) and is altered by emotional states such as stress, maternal left-cradling was interpreted as reflecting right-hemispheric dominance for emotional processing. Whether this phenomenon is unique to human evolution is still in debate. In the present study we followed 44 olive baboon (*Papio anubis*) mothers and their infants in different social groups. We found that a maternal cradling bias exists and is predominantly towards the left in a similar proportion as in humans, but shifts toward a right bias in mothers living in high density groups. The sensitivity of left-cradling to social pressure highlights its potential links with the mother’s stress as reported in humans. Our finding clearly illustrates the phylogenetic continuity between humans and Old-World monkeys concerning this lateralization and its potential links with hemispheric specialization for emotions, inherited from a common ancestor 25–35 million years ago.

## Introduction

In Humans, about 66–72% of mothers prefer to cradle their infants on the left side of their body midline^1^. In other words, mothers hold their infant in their arms, close to their body, positioning its head in their left peri-personal hemispace and support the weight with their left arm^2^. The left-cradling bias persists for at least the first 12 weeks after birth^3^. However, at the human population-level, inanimate objects (i.e., bags) are carried on the right side for the greatest part^4^. However, a pillow adorned with a proto-face is enough to elicit a left-cradling bias in children^5^. This phenomenon has raised lots of questions and studies which have shown that a contralateral relation with handedness is not present. Indeed, although left-side maternal cradling leaves the right hand free for other activities in right-handed mothers, left-handed individuals turn out to also present a left-cradling bias^5^, (but see^6,7^). Besides manual preference, heart position (i.e., soothing sound of heartbeats^8–10^), cultural considerations^11^, or the different activities during which cradling occurs (i.e., rocking, nursing^12–14^) seem not to affect the left-cradling bias. The most consensual theory to date combines visual field and cerebral hemispheric specialization. The “right-hemisphere hypothesis” highlights the posture of a left-cradled baby, as it exposes its face to the left visual field of the mother^15^. Due to the contralateral organization of the human sensory systems, this visual information is mainly projected to the mother’s right brain hemisphere. As the right hemisphere of the brain is specialized in the perception of emotional facial expressions^16–19^, the left-cradling bias would consequently favour the mother’s monitoring of the emotional state of the infant.

In parallel, the left-cradled infant looks at the left side of the mother’s face, which has been described as being the most expressive^7,20,21^. It has been assessed that 3-month-old children identify the attractiveness of a face as well as adults do^22^. According to some authors, this direct access to the mother’s emotional state would then facilitate creating and reinforcing social bonds within the mother baby dyad^15^. Also, it has been shown that left-cradling mothers judge as more attractive a left- rather than a right-facing profile of a human baby (and vice versa for right-cradling ones)^23^. Recent studies^2,5^ pushed the investigations on the left-cradling bias further, showing a relationship with empathy and ill-being in humans. On the one hand, they found the strength of the left-cradling bias to be positively related with the mother’s empathic abilities^2^. Moreover, children cradling a doll on their left display higher mean social ability scores than their peers showing a right-cradling bias^5^.

On the other hand, right-side cradling is associated with higher pre-and postnatal maternal anxiety and depression^24,25^. Maternal depression involves decreased communication within the mother-infant dyad^26^ and a dysfunction of the right brain hemisphere affecting emotional perception. It may therefore be considered as a factor that alters the left-cradling bias^27^. Other studies have found higher stress levels in mothers with a right-cradling bias, than in their counterparts cradling on the left^28^. Stress can immediately impact the infant cradling: under induced physiological stress conditions (identified by a higher blood pressure and heart rate), women hold a human-like doll more on the right^29^. In both 4 and 5-year-old boys and girls, the left-cradling bias is already strongly present when cradling a human infant-like doll, but can be reversed under unfamiliar or stressful stimuli^5^. Affective symptoms can therefore alter left cradling, reflecting a reduced ability to be emotionally involved with the infant.

Facing such a biological phenomenon, a question arises: is this trait specific to humans? Most of the work in other vertebrate species focusing on patterns of lateralization in mother-infant interactions has shown a population-level preference for keeping the mother on the left side in infants^30^. Concerning maternal cradling specifically, which involves holding the infant close to one’s body by using the arms and hands^2^, humans are not the only primate species showing this lateralized behavior, as great apes and monkeys like macaques and baboons also cradle their babies during the first months of life. This behaviour is mainly female-specific and maternal. A left side bias in infant holding has been reported mostly in great apes^8,31,32^ whereas studies in our more distant primate relatives, such as Old or New World monkeys reported no consistent cradling bias^14^. The early onset of left-cradling bias in humans and its presence in great apes suggest an evolutionary continuum of the dominance of the right brain hemisphere for processing social-emotional stimuli^5,33^. The left-cradling bias could thus be the result of biological determinism selected through natural selection rather than an intentional act. Nevertheless, no study to date has investigated the link between a left-cradling bias and the maternal emotional state in non-human primates.

**Figure 1:**
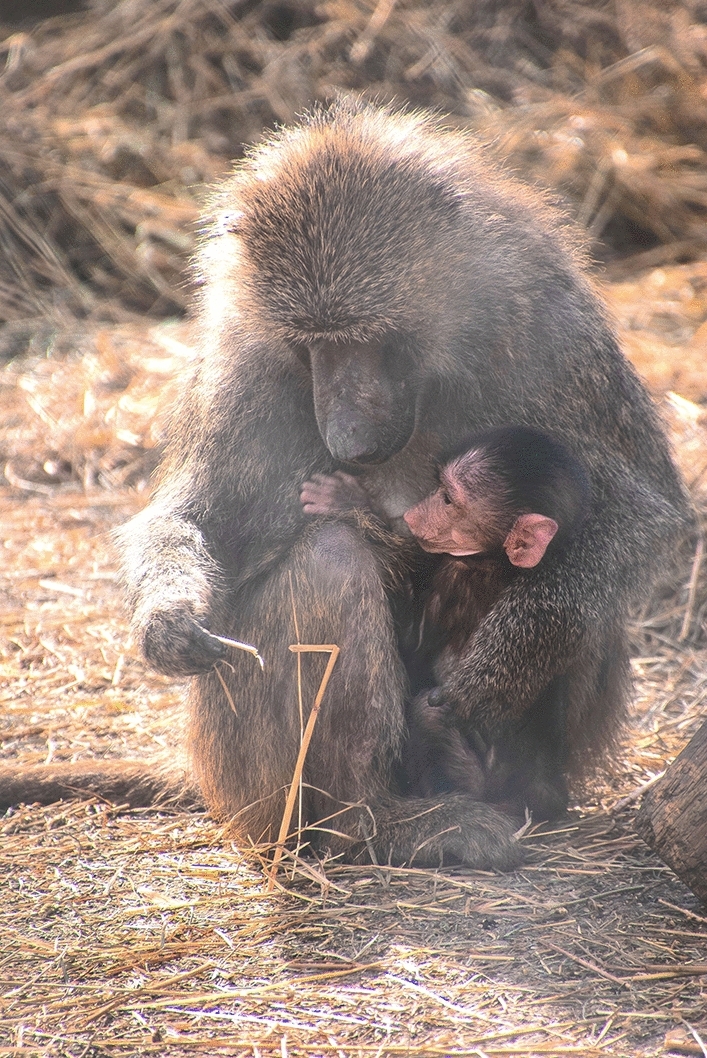
Maternal cradling in an adult female olive baboon. A baboon mother is cradling her baby on her left side. Photograph copyright: Eloïse Disarbois.

In the present study we investigated (1) side-asymmetries in maternal cradling behaviours during the three first months after the infant’s birth in the olive baboon (*Papio anubis*) (see Fig. 1). We also (2) explored its potential relation with the social group density as a marker of social stress*.* We first expect cradling to be lateralized in female olive baboons at the individual level and predict a population-level left-cradling bias similar to humans. As olive baboons organize themselves into very hierarchical social groups, agonistic behaviours, social pressure and thus socially-related stress strongly increase with density^34,35^. If, as in humans, stress is a factor affecting the left-cradling bias, we therefore expect the left-cradling bias to be altered or even reversed in females housed in high densities social groups.

## Results

### Maternal cradling-side bias at individual level

**Figure 2:**
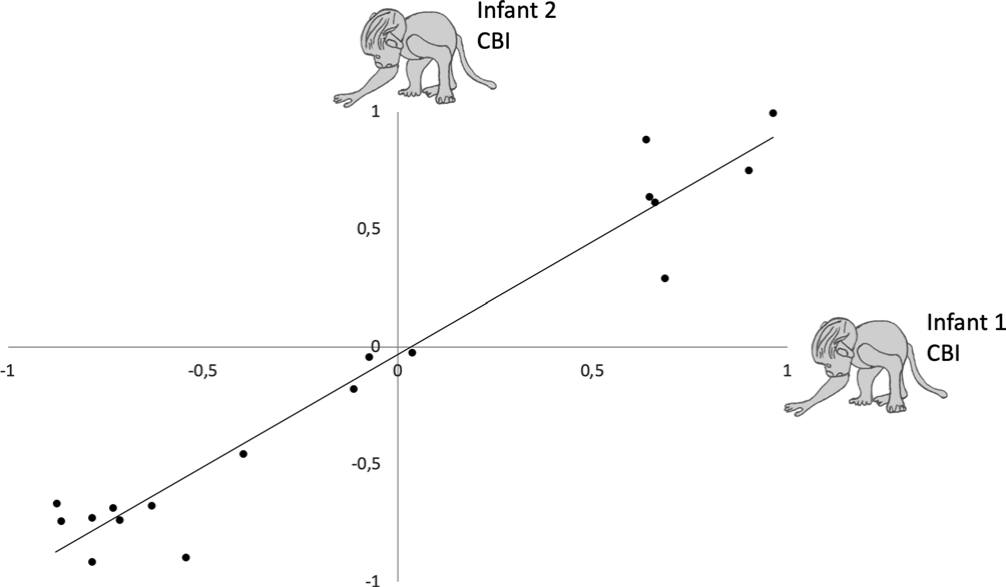
Plot depicting correlation between individual CBI from successive newborns*. *N = 18. The maternal cradling-side bias index (CBI) is the ratio (R − L)/(R + L), where R and L respectively represent the total right and left arm uses. It ranges from − 1 to 1. A negative ratio indicates a left side cradling bias, whereas a positive one indicates a right-cradling bias.

According to *z*-score, 40 mothers were classified as significantly lateralized for maternal cradling (left- or right-sided) while only 4 were considered as ambiguously lateralized. Out of the 18 females that had two pregnancies during the study time, the direction and strength of the individual cradling bias was very stable across infants (Pearson correlation *r*_17_ = 0.97, *P* < 0.0001; see Fig. 2). In fact, 17 kept the same cradling-side bias while only one female switched from an ambiguously lateralized to a left-biased cradling, but both cradling-side bias indices (CBI) had the same negative sign, indicating similar left direction of the cradling-side bias. Neither an infants’ sex or maternal parity were significant predictors of cradling lateralization.

### Maternal cradling-side bias at population level

According to the *z*-score, among the 40 lateralized mothers, 27 cradled significantly their infant on the left side and 13 on their right side. This translated into a significant left-cradling bias at population-level (*X*^2 ^= 4.9, *P* = 0.026; *N* = 40). The calculation of the mean cradling-side bias index score among the total of 44 mothers, M.CBI = − 0.20, SD = 0.65, confirmed the left-cradling bias at a population-level according to a one-sample *t *test (*t*_43_ = − 2.03, *P* = 0.047).

### Effects of social group structures on the population-level cradling-side bias

**Figure 3:**
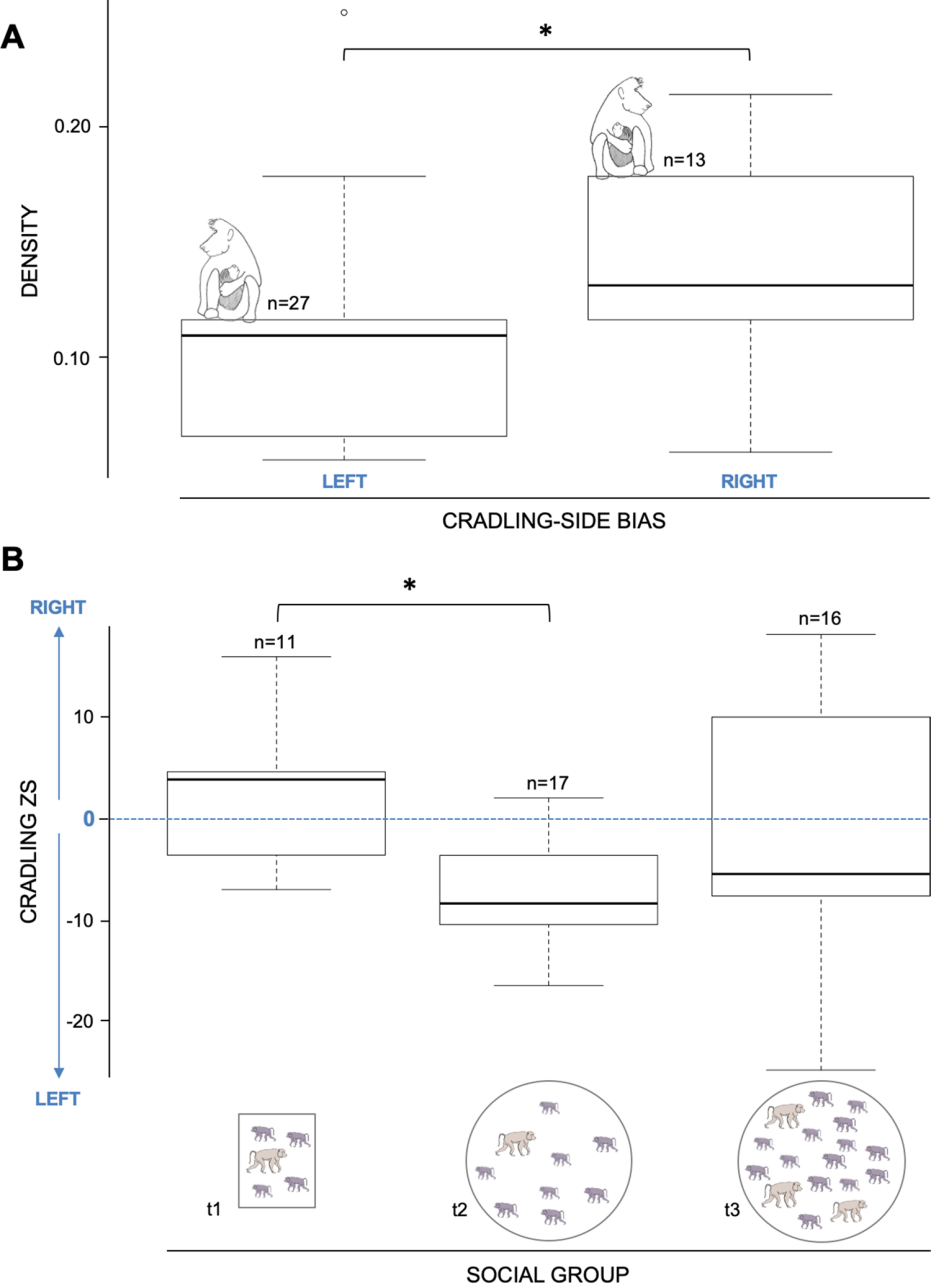
Effect of social density on maternal cradling-side bias. (**A**) Boxplot of the densities of social groups housing *Papio anubis* females (n = 40) regarding their cradling-side bias (i.e., based on z-score and excluding 4 ambiguously lateralized cases). (**B**) Boxplot of the cradling *z*-scores of *Papio anubis* females according to their social group structures: high density mono-male aviaries (t1), low density mono-male parks (t2) and a high density multi-male park (t3). Adult males are in beige and females and juveniles are in purple. The calculation of the *z*-score is based on the total left and right arm uses. It provides the direction of cradling side preference: left (< − 1.96) or right (> 1.96). (*P value < 0.05).

Linear models detected that the cradling *z*-score significantly increased with increasing density (*F*_1,42_ = 5.30; *P* = 0.026), indicating an increase in a right cradling bias. By running a Kruskal–Wallis rank sum test with the cradling-side bias (i.e., based on *z*-score calculation) as a qualitative variable and the density as a quantitative variable, we found that the 27 left cradling mothers lived in significant lower density social groups (mean density = 0.11) than the 13 right cradling mothers (mean density = 0.15; Kruskal–Wallis *X*^*2*^ = 6.14, *P* = 0.013; see Fig. 3A). After running a second linear model with the type of group structures (i.e. mono-male aviaries and parks, multi-male park) as significant predictor of cradling *z*-score (*F*_2,41_ = 4.60; *P* = 0.015), we made a pairwise comparison using *t *tests and a Bonferroni correction. When comparing females housed within mono-male groups but in high densities aviaries (t1) *versus* lower densities parks (t2, see Table 1), we found a strong significant difference (*P* = 0.02). Females housed in mono-male parks cradled mainly on their left side (*z*-score mean = − 7.19; CBI mean = − 0.56), whereas females housed in mono-male aviaries cradled more on their right side (*z*-score mean = 2.36; CBI mean = 0.20). The multi-male park (t3) showed higher variability and was not significantly different from the two other types of group structures (*z*-score mean = − 0.68; CI mean = − 0.08; see Fig. 3).

**Table 1:** Description of the different habitats housing the olive baboon subjects during the study.

Habitat	Aviaries (t1)	Park 1 (t2)	Park 2 (t2)	Park 3 (t2)	Park 4 (t3)
Surface (m^2^)	28	291	291	211	267
Adult males	1	1	1	1	3
Focal mothers	11	6	6	5	16
Group size	5.57 (± 0.78)	19	21	23	38
Density	0.19(± 0.028)	0.07	0.07	0.11	0.14

Habitats include mono-male and multi-male social group structures. Of the 44 focal females 11 were housed in seven aviaries of same surface; the average group size and density are provided with standard deviations. The group sizes of the four parks, where the 33 other females lived, were subject to variations as some individuals died or were moved within the station. The table provides the maximal group sizes and densities observed in these parks during the study time.

## Discussion

In accordance with our predictions, we found a significant left-side maternal cradling bias in an Old-World monkey, the olive baboon (*Papio anubis*) in an almost identical proportion (i.e. 67.45% among the 40 lateralized subjects) to the one usually found in human mothers (64% in the most recent study^2^). This finding, which is consistent with similar evidence from great apes, namely chimpanzee (*Pan troglodytes*) and gorilla (*Gorilla gorilla*)^31,32^ clearly indicates that this lateralization phenomenon is not unique to humans but also present in catarrhines. Previous inconsistent results in macaques and baboons (for a review^14^) are likely due (1) to too small sample sizes (i.e., from 4 to 10 subjects) preventing to draw any conclusions about population-level lateralization, or (2) to the non-consideration of the social density in a macaques’ study including a substantial sample of captive individuals^13^. Interestingly, we also found that the direction and the degree of individual maternal cradling bias is consistent and stable across successive pregnancies within the same mothers. This suggests that, as for humans, left-cradling bias is a robust lateralized behaviour, which might be ultimately related to the same underlying hemispheric specialization process. The drivers of the observed convergence between olive baboons and humans remain unclear. One hypothesis is that left-side maternal cradling might reflect rightward hemispheric specialization for emotions, as has been suggested in humans^2,5,15^, since it favours the mother’s left visual field of view (i.e. right hemisphere processing) for maternal monitoring. Given that a right hemisphere advantage for facial emotional expressions processing has been highlighted not only in humans^16^, but also in other primate species, namely chimpanzees^36^, rhesus monkeys^37^ and baboons^38^, it is not unlikely that a shared hemispheric specialization for emotion might be involved in the manifestation of the left-cradling bias we observed in baboons. However, this latter hypothesis needs to be tested specifically in further investigations. In humans, left-cradled infants look at the left side of the mother’s face, exposing therefore their “right hemisphere” to the most expressive side of the mother’s face^7,20,21,39^. Such typical left-side cradling pattern might in turn trigger a typical neurodevelopment in the cradled infant. It has been shown that individuals who had been cradled on the mother’s right side during infancy showed a significant decrease of the typical left bias for emotional faces compared to left-cradled individuals^40^. It is possible that a reduced exposure to optimal (i.e., left) or suboptimal (i.e., right) emotional information from faces during infanthood (due to a reversed lateral holding position) might have crucial outcomes for the ability to perceive facial emotions later in life and for cascades of other related socio-emotional abilities (thus being beneficial for the child’s later development of such functions). In this regard, based on interlocutory findings linking the cradling-side bias with social attachment abilities^41^ and the presence of autistic traits^42,43^, it has been very recently hypothesized that the typical/atypical holding-side during infancy might be one of the possible early signs of potential neurodevelopmental dysfunctions such as autism spectrum disorders^44,45^. In addition, asymmetries in an infant’s positioning have also been reported in non-primate species that do not carry their babies. In a wide range of marine and terrestrial mammals, juveniles have a strong preference for keeping their mother on their left side, namely in their left visual field^30^. This has prompted previous authors to propose the idea that the right lateralized “social brain” as described in primates has an ancient evolutionary origin. It would be derived from earlier forms of lateralization in vertebrates, namely lateralization in interactions within the mother-infant dyad that promote bonding and thus maximize the infants’ survival. Our results push the potential link between cradling and emotions further by showing that the left-cradling bias may be an evolutive proxy of the maternal emotional state in an old-word monkey, as it is in humans^2,5^.

More importantly, we further found lateralized cradling at the individual-level to be strongly related with demographic densities, and not group size. While mothers living in low density groups show a strong left-cradling bias, mothers living in high density groups show a weaker left-side bias, or even a right bias during cradling. One potential explanation of the sensitiveness in cradling bias to social density might be found in its tight relation with the mother’s social stress. According to this hypothesis, which remains to be tested, the maternal left-cradling bias would be, just like in humans^28,29^, altered by stress, which is in baboons attributed to high social density. Olive baboons organize themselves into very hierarchical social groups^46^. Relationships of dominance result in a strong power struggle. Higher densities increase the occurrence and frequency of conflicts involving severe aggression^34,35^. Such conditions involve higher levels of stress. In humans, whereas left-cradling bias is positively related with empathy^2^ and stronger social abilities^5^, it is negatively related with depression and anxiety^2,24,25^. Maternal depression involves a dysfunction of the right brain hemisphere affecting the perception of emotions and may therefore alter the left-cradling bias^27^, as it is observed under stressful stimuli^28,29^. Higher densities in olive baboon social groups, and thus greater social pressure, may also have a cost for the mothers by mobilizing more attentional and emotional resources in the right hemisphere to the detriment of infant’s lateralized monitoring. In fact, the several social functions, that are particularly recruited in high density groups, mobilize the right side of the brain such as (1) aggressive responses^47^ from dominant females ensuring their rank or, (2) processing conspecific faces^47^ in low-rank females to discern aggressive expressions, and (3) display avoidance or withdrawal behaviors^47^. According to this hypothesis, the socially related stress would thus alter the rightward hemispheric resources allocated to the maternal monitoring and ultimately affect the left-cradling bias. Nevertheless, whether different confounding factors related to social density—other than stress—may explain this variability remains unclear. Unfortunately, no study has investigated the direct link between cradling-side bias and the maternal emotional state in non-human primate species. To test the “stress hypothesis” in non-human primates, further investigations are thus needed and should consider evaluating the potential direct links between cradling-side bias and (1) the stress level of the mother (e.g., cortisol level assessment, induced stress situation), or (2) the hemispheric specialization for emotions (e.g., brain structural correlates using in vivo MRI-imaging, behavioral asymmetry testing related to the lateralization of emotions such as facial expressions or chimeric face processing).

In conclusion, we suggest that the origin of a left-cradling bias may be much older than the origin of humans and even older than hominids and hominoids, dating back to common ancestor of humans and Old-World monkeys about 25–35 million years ago. The convergence of findings for baboons and humans as well as the cradling bias’s sensitiveness to social density opens the road to the hypothesis that a similar hemispheric specialization for emotion may be involved in the maternal cradling lateralization phenomenon and its alteration by stress.

## Methods

### Experimental model and subject details

All our subjects (*Papio anubis*) were born and raised in captivity in the UPS 846 Primate Center of the C.N.R.S. (Rousset, France). We followed 44 adult mothers and 63 juveniles (from 1 day to 3 months), as 17 mothers gave birth to 2 infants and 1 mother gave birth to 3 infants successively during the time frame of the study. Olive baboons live in social groups in large aviaries or parks from 28 to 291 m^2^, (see Table 1). Each enclosure included an inside and an outside area. The 44 mothers lived in 3 different types of habitats and group structures (see Table 1) including (t1) mono-male multifemale structures in high density small social groups (i.e., seven aviaries); (t2) mono-male multifemale structures in low density large social groups (i.e., three parks: 1, 2, 3), and (t3) multi-male multi-female structures in large but high density social group (i.e., one park 4). Our sample of 63 infants includes 33 females and 28 males (the sex of two infants was not identified). At the beginning of the study, 14 females were primiparous and 30 were multiparous. *Ethics* The study was approved by the “C2EA-71 Ethical Committee of Neurosciences” (INT Marseille) under the number APAFIS#13553-201802151547729, and has been conducted at the Station de Primatologie (Rousset-Sur-Arc, France, Agreement C130877). All methods were performed in accordance with the relevant CNRS guidelines and the European Union regulations (Directive 2010/63/EU).

### Procedure of data collection

Among the focal baboons’ mother-infant dyad, we quantified the use of the left arm *versus* the right arm to cradle the infant, regardless of the mother’s postures (see Fig. 1) or of feeding interactions. According to a behavioral sampling procedure, a minimum of 30 occurrences of cradling was collected for each dyad (min = 34, max = 784; mean = 172, SD = 151). We collected data during the three first months after birth, the baby being almost exclusively cradled during this period. After this period, mothers cradle less and less and carry the juveniles on their back or on their belly but without a support by the arm. We counted an occurrence as an independent bout of cradling. Every time the mother stopped cradling her baby—for manipulation (i.e., grooming, play) or to let him get out of the embrace—a cradling period ended; the next cradling was then reported as a new occurrence. Cradling bouts were considered only if both of the mother’s hands were free (i.e., not holding an object in one hand); thus, we assumed there was an independent choice of hands for cradling.

### Statistical analysis

We first determined the direction of cradling-side asymmetry for each mother and each infant by calculating a *z*-score, based on the total left and right arm uses^48^. We then classified each mother as having a left side (*z* ≤  − 1.96) or right side (*z* ≥ 1.96) cradling bias, or behaving ambiguously (− 1.96 < *z* < 1.96). We also quantified the degree of the cradling-side bias for each subject by calculating an individual cradling-side bias index score (CBI) using the formula (R − L)/(R + L). R and L respectively represent the total right and left arm uses^48,49^. A negative value indicates a left side cradling bias, whereas a positive value a right-side bias.

We performed the following statistical analyses: (1) we performed a Pearson correlation test to assess whether direction and strength of the individual cradling bias were stable across offspring or not. We selected the females who had 2 pregnancies successively within our study period, and correlated the mother’s CBI for the first infant with the one for the second infant. (2) We additionally ran a multiple linear regression to test effects of experience (i.e., parity), and infant’s sex as predictors of cradling lateralization (i.e., *z*-score). (3) We tested for a left-cradling bias at a population level by performing a Chi-squared test for given probabilities (0.5, 0.5) comparing the number of mothers cradling their infant on the left side versus on their right side (classification based on *z*-scores). (4) We also tested for a population-level left-cradling bias by calculating the mean cradling-side bias index score, and running a one-sample *t *test. (5) In order to test an effect of social density on the population-level left-cradling bias, we ran a linear model with density as quantitative predictor of cradling *z*-score. We further ran (6) a Kruskal–Wallis rank sum test with the cradling-side bias (i.e., based on *z*-score calculation) as a qualitative variable and the density as a quantitative variable. (7) After running a second linear model with the type of group structures (i.e. mono-male aviaries and parks, multi-male park) as significant predictor of cradling *z*-score, we made a pairwise comparison using *t* tests and the Bonferroni *P *value adjustment method. We checked normality by performing a Shapiro–Wilk Normality test. We used the following RStudio packages: FactoMineR, car, MASS, readxl. All statistics were performed using a conventional level of significance (*P* = 0.05).

## Supplementary information

Supplementary information

## Data Availability

The datasets supporting this article have been uploaded as part of the supplementary material. They will be moved to an external repository upon publication.

## References

[1] Packheiser J, Schmitz J, Berretz G, Papadatou-Pastou M, Ocklenburg S (2019). Handedness and sex effects on lateral biases in human cradling: three meta-analyses. Neurosci. Biobehav. Rev..

[2] Malatesta G, Marzoli D, Rapino M, Tommasi L (2019). The left-cradling bias and its relationship with empathy and depression. Sci. Rep..

[3] Todd BK, Banerjee R (2015). Lateralization of infant holding by mothers: a longitudinal evaluation of variations over the first 12 weeks. Laterality.

[4] Almerigi JB, Carbary TJ, Harris LJ (2002). Most adults show opposite-side biases in the imagined holding of infants and objects. Brain Cogn..

[5] Forrester GS, Davis R, Mareschal D, Malatesta G, Todd BK (2019). The left cradling bias: an evolutionary facilitator of social cognition?. Cortex.

[6] Harris LJ, Almerigi JB, Kirsch EA (2000). Side preference in adults for holding infants: contributions of sex and handedness in a test of imagination. Brain Cogn..

[7] Vauclair J, Donnot J (2005). Infant holding biases and their relations to hemispheric specializations for perceiving facial emotions. Neuropsychologia.

[8] Salk L (1960). The effects of the normal heartbeat sound on the behavior of the new-born infant: implications for mental health. World Mental Health.

[9] Salk L (1973). The role of the heartbeat in the relations between mother and infant. Sci. Am..

[10] Todd B, Butterworth G (1998). Her heart is in the right place: an investigation of the ‘heartbeat hypothesis’ as an explanation of the left side cradling preference in a mother with dextrocardia. Early Dev. Parent..

[11] Richards JL, Finger S (1975). Mother-child holding patterns: a crosscultural photographic survey. Child Dev..

[12] Bundy RS (1979). Effects of infant head position on sides preference in adult handling. Infant Behav. Dev..

[13] Tomaszycki M, Cline C, Griffin B, Maestripieri D, Hopkins WD (1998). Maternal cradling and infant nipple preferences in rhesus monkeys (*Macaca mulatta*). Dev. Psychobiol..

[14] Hopkins WD (2004). Laterality in maternal cradling and infant positional biases: implications for the development and evolution of hand preferences in nonhuman primates. Int. J. Primatol..

[15] Manning JT, Chamberlain AT (1991). Left-side cradling and brain lateralization. Ethol. Sociobiol..

[16] Bryden, M. P. & Levy, R. G. in *Neuropsychology of Human Emotion* (eds Heilman, K.M. & Satz, P.) (Guilford Press, New York, 1983).

[17] Best CT, Womer JS, Queen HF (1994). Hemispheric asymmetries in adults’ perception of infant emotional expressions. J. Exp. Psychol. Hum. Percept. Perform..

[18] Gainotti G (2019). Emotions and the right hemisphere: can new data clarify old models?. Neuroscientist.

[19] Prete G, Capotosto P, Zappasodi F, Tommasi L (2018). Contrasting hemispheric asymmetries for emotional processing from event- related potentials and behavioral responses. Neuropsychology.

[20] Sackeim HA, Gur RC, Saucy M (1978). Emotions are expressed more intensely on the left side of the face. Science.

[21] Lindell A (2018). Lateralization of the expression of facial emotion in humans. Progr. Brain Res..

[22] Langlois JH, Ritter JM, Roggman LA, Vaughn LS (1991). Facial diversity and infant preferences for attractive faces. Dev. Psychol..

[23] Malatesta G, Marzoli D, Tommasi L (2020). Keep a left profile, baby! the left-cradling bias is associated with a preference for left-facing profiles of human babies. Symmetry.

[24] de Château P (1983). Left-side preference for holding and carrying newborn infants: parental holding and carrying during the first week of life. J. Nerv. Ment. Dis..

[25] Vauclair J, Scola C (2009). Infant-holding biases in mothers and affective symptoms during pregnancy and after delivery. Infant Child Dev..

[26] Sieratzki JS, Woll B (2002). Neuropsychological and neuropsychiatric perspectives on maternal cradling preferences. Epidemiol. Psychiatr. Soc..

[27] Weatherill RP, Almerigi JB, Levendosky AA, Bogat GA, Eye A, Harris LJ (2004). Is maternal depression related to side of infant holding?. Int. J. Behav. Dev..

[28] Reissland N, Hopkins B, Helms P, Williams B (2009). Maternal stress and depression and the lateralization of infant cradling. J. Child Psychol. Psychiatr..

[29] Suter SE, Huggenberger HJ, Schächinger H (2007). Cold pressor stress reduces left cradling preference in nulliparous human females. Stress.

[30] Karenina K, Giljov A, Ingram J, Rowntree VJ, Malashichev Y (2017). Lateralization of mother–infant interactions in a diverse range of mammal species. Nat. Ecol. Evol..

[31] Manning JT, Chamberlain A (1990). The left-side cradling preference in great apes. Anim. Behav..

[32] Manning JT, Heaton R, Chamberlain AT (1994). Left-side cradling: similarities and differences between apes and humans. J. Hum. Evol..

[33] Bourne VJ, Todd BK (2004). When left means right: an explanation of the left cradling bias in terms of right hemisphere specializations. Dev. Sci..

[34] Aureli F, Van Panthaleon Van Eck CJ, Veenema HC (1995). Long-tailed macaques avoid conflicts during short-term crowding. Aggress. Behav..

[35] Campbell CJ (2006). Lethal intragroup aggression by adult male spider monkeys (*Ateles geoffroyi*). Am. J. Primatol..

[36] Morris RD, Hopkins WD (1993). Perception of human chimeric faces by chimpanzees: evidence for a right hemisphere advantage. Brain Cogn..

[37] Guo K, Meints K, Hall C, Hall S, Mills D (2009). Left gaze bias in humans, rhesus monkeys and domestic dogs. Anim. Cogn..

[38] Wallez C, Vauclair J (2011). Right hemisphere dominance for emotion processing in baboons. Brain Cogn..

[39] Hendriks AW, van Rijswijk M, Omtzigt D (2011). Holding-side influences on infant’s view of mother’s face. Laterality.

[40] Vervloed MPJ, Hendriks AW, van den Eijnde E (2011). The effects of mothers’ past infant-holding preferences on their adult children’s face processing lateralisation. Brain Cogn..

[41] Malatesta G, Marzoli D, Piccioni C, Tommasi L (2019). The relationship between the left-cradling bias and attachment to parents and partner. Evol. Psychol..

[42] Pileggi L-A, Malcolm-Smith S, Solms M (2015). Investigating the role of social-affective attachment processes in cradling bias: the absence of cradling bias in children with Autism Spectrum Disorders. Laterality.

[43] Fleva E, Khan A (2015). An examination of the leftward cradling bias among typically developing adults high on autistic traits. Laterality.

[44] Malatesta G, Marzoli D, Tommasi L (2020). The association between received maternal cradling and neurodevelopment: is left better?. Med. Hypotheses.

[45] Malatesta G, Marzoli D, Apicella F, Abiuso C, Muratori F, Forrester GS, Vallortigara G, Scattoni ML, Tommasi L (2020). Received cradling bias during the first year of life: a retrospective study on children with typical and atypical development. Front. Psychiatry.

[46] Johnson JA (1987). Dominance rank in juvenile olive baboons, *Papio anubis*: the influence of gender, size, maternal rank and orphaning. Anim. Behav..

[47] Vallortigara G, Rogers LJ (2005). Survival with an asymmetrical brain: advantages and disadvantages of cerebral lateralization. Behav. Brain Sci..

[48] Meguerditchian A, Molesti S, Vauclair J (2011). Right-handedness predominance in 162 baboons (*Papio anubis*) for gestural communication: consistency across time and groups. Behav. Neurosci..

[49] Meguerditchian A, Vauclair J, Hopkins WD (2013). On the origins of human handedness and language: a comparative review of hand preferences for bimanual coordinated actions and gestural communication in nonhuman primates: on the origins of human handedness and language. Dev. Psychobiol..

